# Effect of mitomocin C in eosinophilic nasal polyposis, in vivo: concentration of IL5 and GM-CSF, RT-PCR

**DOI:** 10.1016/S1808-8694(15)30032-X

**Published:** 2015-10-19

**Authors:** Mirian Cabral Moreira de Castro, Evaldo Assunção, Mariana Moreira de Castro, Ricardo Nascimento Araújo, Roberto Eustáquio Guimarães, Flávio Barbosa Nunes

**Affiliations:** a MSc Surgery Student – Medical School - UFMG – Professor at the School of Medical Sciences, preceptor of Medical Residence – UFMG University Hospital and Santa Casa de Misericórdia Hospital.; b Associate Professor, assistant Professor – Department of Parasitolgy - Universidade Federal de Minas Gerais.; c Otolaryngology resident of the University Hospital - UFMG.; d PhD in parasitology - UFMG, Post-doctorate student in parasitology - UFMG.; e Associate Professor, Assistant Professor of the Department of Ophthalmology, Otolaryngology and Speech Therapy - Universidade Federal de Minas Gerais.; f MSc in Otolaryngology by the Paulista School of Medicine, Preceptor of the Medical Residence at the University Hospital - UFMG. Department of Ophthalmology, Otolaryngology and Speech Therapy of the Universidade Federal de Minas Gerais. Prof. Evaldo Nascimento Laboratory of Immunology.

**Keywords:** nasosinusal polyposis, eosinophils, mitomycin C, interleukin 5, GM-CSF

## Abstract

Eosinophilic nasosinusal polyposis is a chronic inflammatory infection with elevated infiltration of eosinophils, which presents high rate of recurrence after surgical treatment. The continuous inflammatory process that leads to the formation of polyps requires constant clinical treatment. Contributing to the maintenance of eosinophilia are cytokines IL5 (interleukin-5) and GM-CSF (granulocyte macrophages colony-stimulating factor), which show up in elevated concentrations. These oligoproteins diminish the rate of apoptosis and prolong the survival of eosinophils.

**Aim:**

By diminishing these cytokines, the action of Mitomycin C (MMC), an antineoplasic drug which inhibits the synthesis of DNA, was studied. In a recent study the power of this drug to cause apoptosis in eosinophils, in vitro, of nasal polyps was verified.

**Methodology:**

A biopsy of the nasal polyps was undertaken in 15 patients carriers of eosinophilic nasosinusal polyposis 24 hours after applying 0.5 mg/ml of MMC during five minutes. RT-PCR (reverse transcription of polymerase chain reaction) for IL5 and GM-CSF was the method used to obtain the results.

**Results:**

The comparison of the results of GM-CSF pre- and post-application of MMC, when the paired T-test was used, showed p=0.041 and for IL5 we found p<0.001.

**Conclusion:**

Topic use of MMC in patients with eosinophilic nasosinusal polyposis shows statistically significant reduction for GM-CSF and significant and important reduction for IL5.

## INTRODUCTION

**Figure 1:**
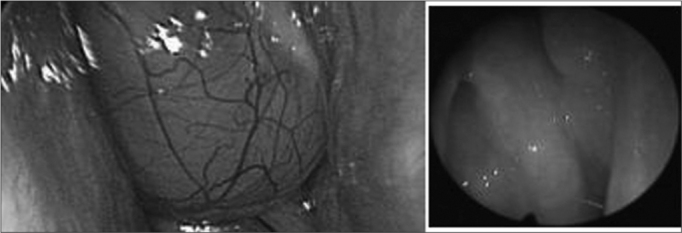
Polyps in an eosinophylic polyposis patient; endoscopic view.

Naso-Sinus Polyposis (NSP) – eosinophylic NSP – is a chronic proliferative inflammatory disease that affects the nasal and nasal sinuses mucosa, and is characterized by a benign polypoid degeneration (Figure 1). It is usually bilateral, has its incidence peak on the fourth decade of life and affects 2.7% of the population, with a 2.2:1 male/female ratio. It is strongly associated to asthma; 1/3 of eosinophilic NSP patients have asthma, therefore, the complete exam of the respiratory tract should be a routine. It is also related to NAER (non-allergic eosinophilic rhinitis), aspirin intolerance, alergic fungal sinusitis and Churg-Strausss syndrome^1^, ^2^.

**Figure 2:**
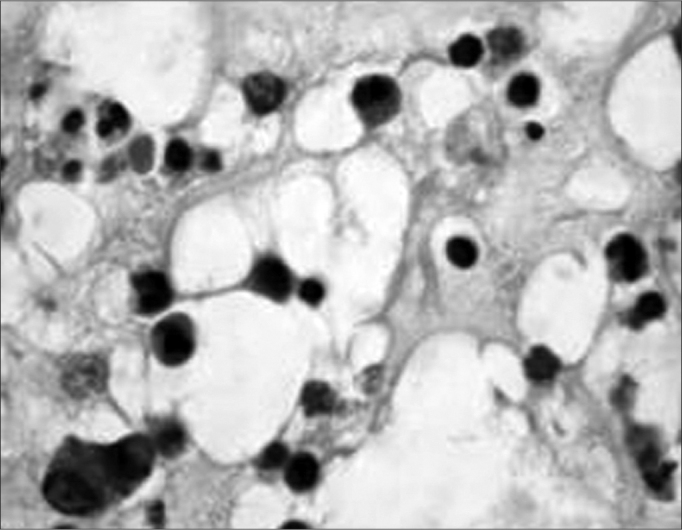
Histology cross-section of an eosinophylic polyp. Stroma bearing eosinophils, lymphocytes and edema.

They are Histologically characterized by eosinophilic granulocytes of which the pathogenic mechanism is not entirely known. They also bear morphologic alterations such as nasal membrane hyperplasia, irregularity of gland distribution, squamous metaplasia and edema (Figure 2)^3^, ^4^.

Although it has been related to IgE mediated hypersensitivity, or nasal allergy, studies have shown that allergy is only one possible cause or a contributing factor^5^, ^6^, ^7^. Eosinophil and polyp structural cells secrete cytokines that maintain the inflammation process and the Eosinophil build up constant. 8 Cytokines such as IL5 and GM-CSF increase eosinophil survival and prolong their presence within the polypoid tissue, reducing the apoptosis rates of these cells^9^, ^10^, ^11^, ^12^.

Eosinophils express CD40 receptor for this GM-CSF and CD40L autocrine production and they are expressed by T CD4 cells that are present in eosinophilic NSP^13^.

The high IL5 concentration found on eosinophilic NSP suggests an autocrine effect for this cytokine in eosinophil activation, there still is a strong relation between IL5 and ECP (eosinophilic cationic protein)^10^, ^14^.

This cytokine increase is related to the genetic aspect of the NSP eosinophilic cells^5^.

Most of the treatment available is based on the use of systemic steroids for a short duration and topical steroid for a longer period, besides surgery^15^.

MMC is an antineoplastic agent produced by the Streptomyces caespitosus that has been used as antiproliferative agent in the 0.4mg/ml concentration. When used after surgery, its action on fibroblasts leads to a reduction in scar tissue formation. It has a time-dependent action e its application for 5 minutes has a measurable effect on cell morphology and proliferation for up to 36 days in vitro16. MMC mechanism of action is based on the selective induction of DNA synthesis, recombination and exchange of “sister” chromatin. Its association with other anti-tumoral drugs leads to an increase in its cytotoxic effect by increasing apoptosis induction^17^, ^18^, ^19^.

Crosara et al. (2004) showed that MMC is efficient in inducing the apoptosis of eosinophils present in the stroma of eosinophilic nasal polyps in vitro. Paired studies with nine cultivated samples were carried out. The polyps were treated with 0.4mg/ml for five minutes and were dyed with hematoxylin eosine^15^.

In topical use, this drug has been used in ophthalmology, otology and laryngology without systemic toxicity, thus being considered safe. It does not cause bleeding, necrosis or infection when used in external acriocistorhinostomy or permanent eyelash lesions in concentrations of up to 1mg/ml^16^, ^18^, ^20^, ^21^, ^22^, ^23^, ^24^, ^25^, ^26^, ^27^.

This study aims at assessing MMC action, in vivo, in patients with eosinophilic NSP through IL5 and GM-CSF analysis, pre and 24hs post application, using RT-PCR (reverse transcription in polymerase chain reaction).

## MATERIALS AND METHODS

This investigation was made up of eosinophilic NSP patients who agreed to participate in the study, after approval by the Research Ethics Committee. The variables were made up of GM-CSF and IL5 profiles. 15 patients were studied, 6 women and 9 men. Their ages varied from 30 to 57 years.

As inclusion criteria, we used: presence of eosinophilic NSP and no aspirin intolerance. Eosinophilic polyps were considered when tissue eosinophylia as greater then 30% among inflammatory cells, determined by finding at least four eosinophils in each large magnification microscopic field^28^. Patients with previous history of aspirin intolerance, who underwent previous surgery or severe steroid-dependent asthma patients were excluded.

The patients selected underwent MMC application on the right nasal cavity polyps in the concentration of 0.5mg/ml in a cotton ball wet with 1 ml of the drug, where it remained for five minutes. The left nasal cavity was the control-group, considered as pre-use.

PCR biopsies after 24h of MMC use were made in the right nasal cavity and, following that, in the left nasal cavity as well. Tissues collected had average size of 10mm, were then immediately taken to the University Immunology lab where they remained stored at 80 degrees below zero.

The cytokines profile was checked on the polypoid tissue through reverse transcription in polymerase chain reaction (RT-PCR) for IL5 and GM-CSF.

Primer sequence used in the PCRs

Gene Forward Reverse GM-CSF AGAAATGTTTGAC-CTCCAGGA TTGCACAGGAAGTTTCCG IL 5 CTGAGGAT-TCCTGTTCCTGT CAACTTTCTATTATCCACTC

**Figure 3:**
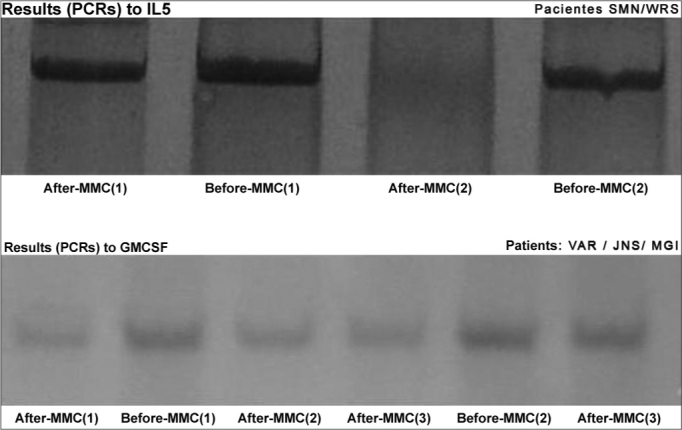
IL5 and GM-CSF PCR results before and after MMC.

The PCR products were analyzed by electrophoresis in silver dyed 8% polyacrylamide gel. The gels were photographed using the AlphaDigiDoc 1201 photo-documentation system, and the PCR resulting bands for each sample were analyzed by densitometry, using the AlphaEaseFC software version 3.3.0 (Alphalnnotech) (Figure 3). This software calculates the IDV - Integrated Density Value for each band, and it is defined as the pixel intensity in the region outlined by the band, subtracted from the pixel intensity of the bottom color of the gel band.

## RESULTS

**Figure 4:**
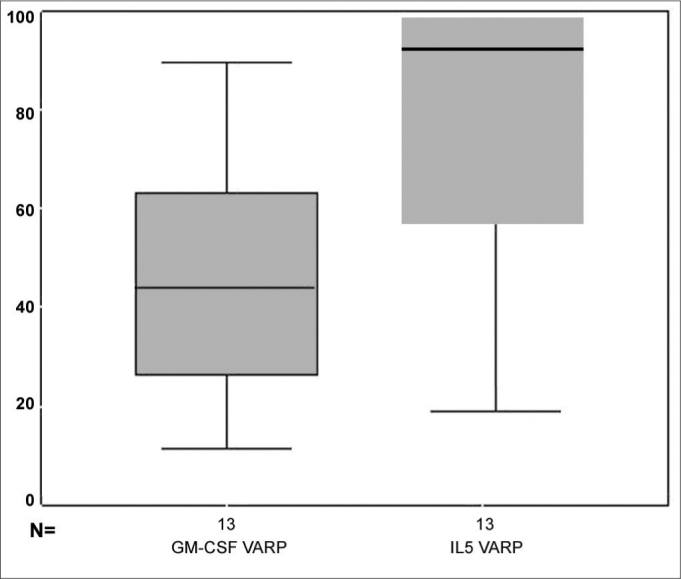
box-plot chart showing the IL5 values before and after MMC.

Regarding the values obtained for IL5, in Figure 4 we see the box-plot distribution chart before and after MMC use. We see that 75% (upper part of the box) of IL5 values after MMC are below 3,000 while almost 100% (lower chart line before) of the IL5 pre MMC values are above this value.

Comparing the IL5 average pre and post, using the paired t test we saw a p<0.001, indicating important statistical significance.

**Figure 5:**
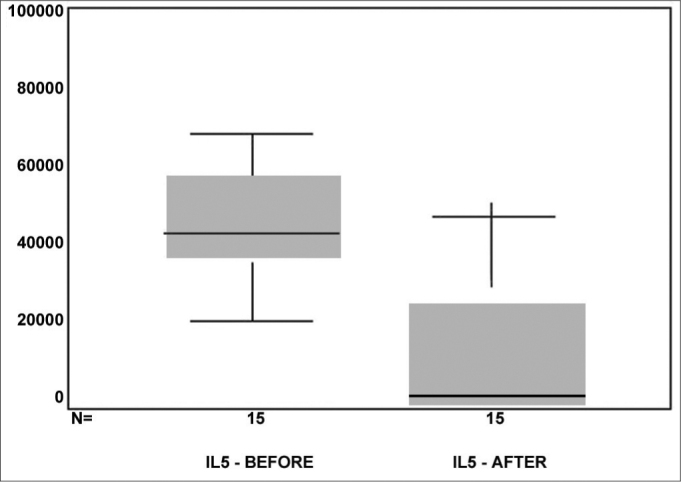
box-plot chart showing the GM-CSF values before and after MMC.

In relation to the GM-CSF values on Figure 5, we see the box-plot chart of the GM-CSF pre and post MMC application distribution values and notice that 75% (upper box) of GM-CSF values are below 15,000 while only 50% of the GM-CSF pre MMC values are below this threshold (middle line of the pre MMC chart). The average pre and post GM-CSF comparison using the paired t test, showed a p =0.041. Thus, the observed difference does bear statistical significance.

**Figure 6:**
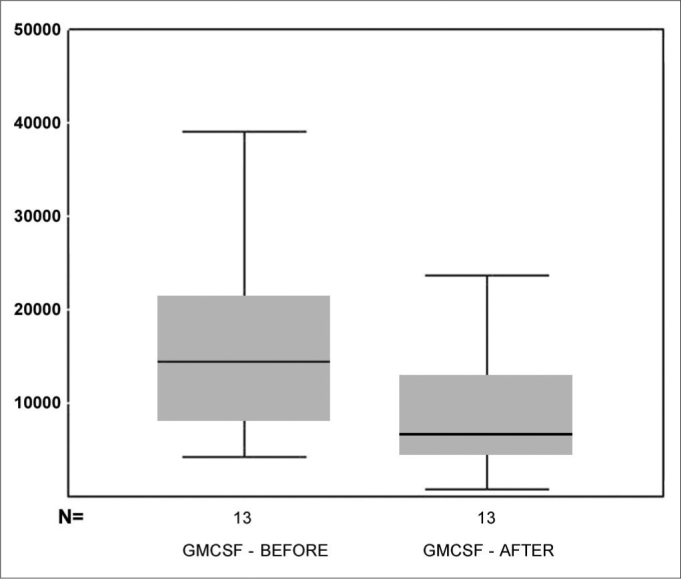
box-plot chart showing the GM-CSF and IL5 reduction percentage.

Figure 6 shows GM-CSF and IL5 percentage reduction in the distribution values box-plot chart. We can see that practically over 75% of patients had an IL5 reduction above 60%, because the box line is somewhat below this value and that 75% of the patients had a GM-CSF reduction below 60%, because the upper box line is somewhat below this value.

Table 1 shows the Pearson's correlation coefficient for IL5 and CM-CSF variation percentage comparison with the continuous variables.

**Table 1:** Correlation and determination coefficient among the continuous variables.

	R	r^2^	p
% IL5 reduction			
Age	0,28	0,045	0,446
Eosinophils	0,112	0,013	0,691
GMCSF reduction	0,039	0,002	0,899
% GMCSF reduction			
Age	0,125	0,016	0,683
Eosinophils	0,174	0,030	0,570
IL5 reduction	0,039	0,002	0,899

No statistically significant correlation was observed, in other words, the IL5 and GM-CSF percentage variation reduction was not followed suit by the variation of any of the other variables.

## DISCUSSION

The participation of polymorph nuclear cells in the nasal mucosa epithelial interface and in other nose diseases is well understood. The increase in cytokine concentrations proves its inflammatory nature. Eosinophils are sources of interleukins that bear autocrine and modulator effect on the function of other cells^14^. The continuity of the inflammatory response seen in the eosinophylic NSP is related to cytokines such as IL5 and GM-CSF which increase survival and reduce the eosinophil apoptosis rate^7^, ^8^, ^10^, ^11^, ^12^.

Therefore, they regulate proliferation and cell activation, broadening local immune response and polyp formation. Eosinophils express IL5 in all eosinophylic NSP and, approximately 30% of the eosinophils have genetic expression for GM-CSF, with evidences of autocrine effect for these citokines^14^. The eosinophil clearance would be a mechanism to reduce the inflammatory process. The cytokine production is related to the tissue cell phenotype, and numerous studies show the key participation of IL5 and GM-CSF in maintaining the inflammatory activity. Therefore, blocking these cytokines would be a therapeutic way to improve this condition. One single MMC application for five minutes has a measurable effect on cell morphology and proliferation for up to 36 days in vitro^16^.

Crossara et al. Showed that MMC is efficient in inducing eosinophil apoptosis when these are present in the stroma of eosinophylic nasal polyps. A paired study with nine cultivated samples was carried out and we observed that the MMC treated cultures presented 12-hour apoptosis rate significantly higher in relation to the control group (p<0.001)^15^.

MMC proved to have inhibitory action on DNA synthesis, inhibiting the proliferation of many cell lines. Kim et al. showed that its anti human-cultivated-fibroblasts effect may be mediated not only by the antiproliferative action, but also by the degree of apoptosis induced^21^.

Our goal in this study was to show MMC action in reducing the concentration of major cytokines that act in maintaining the eosinophylic NSP inflammatory process. Such study has not yet been found in the medical literature.

The paired t test showed that the post MMC GM-CSF values were significantly lower than the pre values (p=0.041). Comparing IL5 values pre and post MMC, we found one important reduction: the medication was efficient in reducing the concentration of these substances. Comparing the percentage with the other variables, we did not observe any variable that would present significant correlation.

MMC may be an option in the pos-surgical treatment of eosinophylic NSP. Further studies are necessary to better use this drug against this disease.

## CONCLUSION

As to the action of MMC over the main cytokines involved in NSP inflammatory process and in relation to the age and percentage of eosinophils, we concluded that:

1. The IL5 post MMC use values were lower than pre MMC use, with important statistical significance;
2. The GM-CSF post MMC use values were lower than pre MMC use, with statistical significance.
